# The Use of Orientation Templates and Free-Hand Implant Insertion in Artificial Mandibles—An Experimental Laboratory Examination in Fifth-Year Dental Students

**DOI:** 10.3390/dj6030043

**Published:** 2018-09-01

**Authors:** Matthias C. Schulz, Lena Rittmann, Ursula Range, Günter Lauer, Dominik Haim

**Affiliations:** 1 Department of Oral and Maxillofacial Surgery, Faculty of Medicine Carl Gustav Carus, Technische Universität Dresden, Fetscherstr. 74, D-01307 Dresden, Germany; rittmann.lena@googlemail.com (L.R.); Guenter.Lauer@uniklinikum-dresden.de (G.L.); Dominik.Haim@uniklinikum-dresden.de (D.H.); 2 Institute for Medical Informatics and Biometry, Faculty of Medicine Carl Gustav Carus, Technische Universität Dresden, Blasewitzer Str. 86, D-01307 Dresden, Germany; Ursula.Range@tu-dresden.de

**Keywords:** dental education, dental implants, free-hand implant insertion, orientation template, thermoforming

## Abstract

Implant dentistry is a growing field in the education of undergraduate dental students. The present laboratory study evaluates factors which may potentially influence the accuracy of free-hand implant insertion and the use of an orientation template. After three-dimensional planning using coDiagnostiX^TM^, orientation templates, including sleeves for the pilot-drill in regions 41 and 45, were manufactured by thermoforming. Sixty-one fifth year dental students inserted one implant using the orientation template and another implant free-hand in an artificial mandible. Information regarding age, sex, handedness, education, and the time required for implant insertion were recorded. Subsequently, the mandibles were scanned using cone-beam-computed tomography and the accuracy of the implant position was assessed, while statistical analysis followed. The free-hand implant insertion resulted in a distal deviation of −1.34 ± 5.15° and a mesial mismatch of 0.06 ± 0.79 mm at the artificial bone level compared to the sleeves. When using the orientation templates, the deviation decreased to −0.67 ± 3.48° and a distal mismatch of −0.22 ± 0.62 mm was achieved. The difference was statistically significant for the mismatch (*p* < 0.049). Regarding the limitations of our study, it could be said that the accuracy level achieved by dental undergraduates using implant placement with orientation templates is comparable to that in other studies.

## 1. Introduction

In recent decades, implant dentistry is fast becoming an emerging discipline in the area of oral rehabilitation. Currently, the provision of implant-borne crowns, as well as fixed and removable partial dentures, is considered as an established treatment procedure.

In addition to the correct surgical procedures, accurate planning of the implant position regarding anatomical structures (e.g., mandibular canal, maxillary sinus), and its relation to the adjacent teeth and the aesthetic outcome is considered to be a crucial factor for sufficient osseointegration and long-term success [1].

The free-hand technique has been said to display adequate success rates but has shortcomings regarding prosthetic planning and handling of poor bone quality [2]. Regarding the determination of the implant position, there exists a widespread variety ranging from simply guiding the pilot drill, to full-guided preparation of the implant cavity and guided implant insertion [1]. It could be shown that virtual planned and three-dimensional guided implant insertion is significantly superior in accuracy compared to free-hand implant insertion [3,4]. Those findings did apply even for experienced surgeons [5]. Currently, common techniques used to produce drilling templates are the thermoforming, cold-curing acrylic, and three-dimensional plotting techniques [1,6,7]. Variances in the different techniques to manufacture the guides were observed to have a statistically significant influence on the achieved accuracy [1]. Despite the differences, it was stated that there might be no clinical relevance as the deviations were in the range of decimillimeters [1].

Application of the full-guided implant insertion is considered more expensive than free-hand insertion [6]. Moreover, the use of implant guides may sometimes be limited due to reduced mouth opening [8]. Despite higher costs and increased time consumption when using drill guides, advantages were seen regarding an improved outcome and reproducible quality through using this method [2].

Recently, it has been assessed that quality-controlled training is correlated to patients’ safety [9]. Furthermore, Lang et al. and Mattheos et al. stated that implant dentistry should be a part of the curriculum for dental students [10,11]. But not only from the dental education consensus workshop participants’ point of view does a broadening of practical education to include dental implants seem desirable—a recent survey among students of the Dental School of the University of Barcelona yielded the undergraduates’ request for extended practical training in implant dentistry [12]. In order to follow these recommendations, lectures and laboratory courses on implant dentistry have been established in our dental school for several years. As the placement of dental implants is a surgical procedure related with potential risks and complications for the patient, undergraduates need to be trained on the insertion of dental implants before actually placing implants in patients. Thus, a laboratory course teaching the insertion of dental implants in artificial mandibles was included in the curriculum of our dental school, and is still being offered today. In a skills test, Dimitrijevic et al. observed that a majority of undergraduate dental students had problems in estimating distances and depths [13]. This was likely to cause difficulties in determining the correct position or correct distance to the adjacent teeth when placing a dental implant. In order to provide the undergraduates support in finding the pre-planned position and angulation of the implant, orientation templates were given, which allowed the instructors to give students feedback on the accuracy of their implant insertions. This kind of template is frequently used by prosthodontists to determine the desired implant position and angulation. To the best of our knowledge, there is currently scarce literature regarding practical training in implant dentistry for undergraduates.

The aim of the present study was to evaluate the influence on the accuracy of the implant insertion using an orientation template manufactured by thermoforming which determined the position of the pilot drill. It was hypothesized that the application of a template determining the position of the implant would lead to higher accuracy compared with free-hand implant insertion, considering the desired implant position and implant angulation would be more time-effective in inexperienced participants. Thus, a group of undergraduate dental students was examined, and individual aspects, such as age, sex, potential prior professional education, or handedness were also assessed.

## 2. Materials and Methods 

All subjects gave their informed consent for inclusion before they participated in the study. The study was conducted in accordance with the Declaration of Helsinki, and the protocol was approved by the Independent Ethical Review Board of the Technische Universität Dresden, Dresden, Germany, on 8 March 2017 (IRB00001473; project number: EK 116032017).

**Figure 1:**
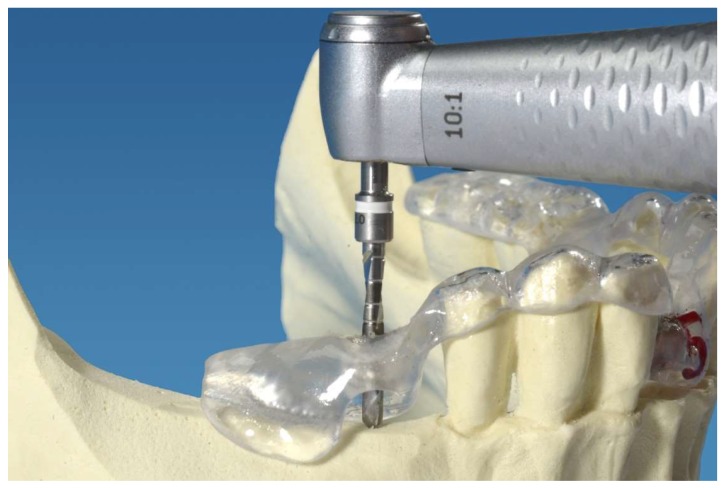
Orientation template fixed on the mandibular model. The pilot cavity in the region of tooth 45 was performed with a 2.0 mm pilot drill.

The planning and manufacturing of the orientation templates was performed according to clinical standards by an experienced dental laboratory technician and an oral surgeon specializing in dental implantology. First, an alginate impression of a partial edentulous plastic mandible model without a mucosa mask (Mandibula Typ A, GOS^®^ GmbH, Northeim, Germany) was performed. A plaster cast using type IV plaster (Excalibur, Dr. Böhme & Schöps Dental GmbH, Goslar, Germany) was manufactured. Next, an X-ray splint was produced by thermoforming using a plastic sheet (Erkodur 1.5 mm; Erkodent GmbH, Pfalzgrafenweiler, Germany) fused to a refFIX^TM^ disk containing three titanium pins of 2 mm diameter (RefPin, IVS Solution AG, Chemnitz, Germany). A cone-beam computed tomography (CBCT, Accuitomo, J. Morita Corporation, Osaka, Japan) was performed with the X-ray splint fixed onto the mandible model. The following parameters were applied: tube voltage, 60.0 kV; current, 3 mA; exposure time, 17.5 s; gantry angle, 0.0°; field of view, 80 × 80 × 80 mm; and voxel size, 0.160 mm. The obtained data was transferred into Digital Imaging and Communications in Medicine (DICOM) files and imported to the three-dimensional planning software, coDiagnostiX^TM^ (Dental Wings, Chemnitz, Germany). The implants were then positioned according to clinical guidelines considering the bone height, bone width, distance, and angulation of the adjacent teeth and, in the premolar region, the distance to the mental foramen. Based on the data, five surgical orientation templates containing a sleeve for the pilot drill each in the region of tooth 41 and tooth 45 were manufactured using the gonyX^®^ device (Institute Straumann AG, Basel, Switzerland). The sleeves (3D Diagnostix Incorporation, Boston, MA, USA) had an inner diameter of 2.0 mm and an outer diameter of 3.0 mm, and were 4.0 mm in length. The mandibular model with the fixed orientation template is shown in Figure 1.

The implants used in the study were dummy implants, according to the Xive^®^ S Plus implant (Dentsply Sirona Implants, Mannheim, Germany). The implant inserted in the front region had a diameter of 3.0 mm and a length of 13 mm. The implant inserted in the premolar region measured 3.8 mm in diameter and 11 mm in length.

As a study population, dental students in their fifth year participating in a required training course in dental implantology were enrolled. Prior to the start of the study, all participants gave their written informed consent. All students completed a questionnaire which requested information regarding age, sex, handedness, potential prior professional education, and their current year of training. The professional education question was classified into professions requiring high manual fine motor skills (e.g., surgeon, porcelain painter), medium fine motor skills (e.g., electrical engineering technicians, chemist) and professions where fine motor skills were not required (e.g., mathematician, office worker). Furthermore, it was asked whether the dental students had already participated in an implant training course and if so, how many times. Following a theoretical introduction regarding the used implant system, all participants were asked to insert one implant into the region of tooth 41 without a guide, and one implant in the region of tooth 45 applying the guide for the pilot drill without a drill stop. The participants were supervised by experienced implantologists having inserted more than 300 dental implants (G.L., M.C.S) [14]. A surgical motor (Frios Unit S/i, W&H Dentalwerk Bürmoos GmbH, Bürmoos, Austria) and the Xive^®^ surgical kit (Dentsply Sirona Implants, Mannheim, Germany) were used. All implant cavities were prepared without irrigation according to the protocol required by the manufacturer. The TempBase^®^ was removed and the appropriate cover screw was inserted. The time taken to insert each implant was recorded, and subsequently, three-dimensional scans using the CBCT were obtained from all mandible models with the appropriate orientation template fixed to the model according to the pre-interventional scan.

**Figure 2:**
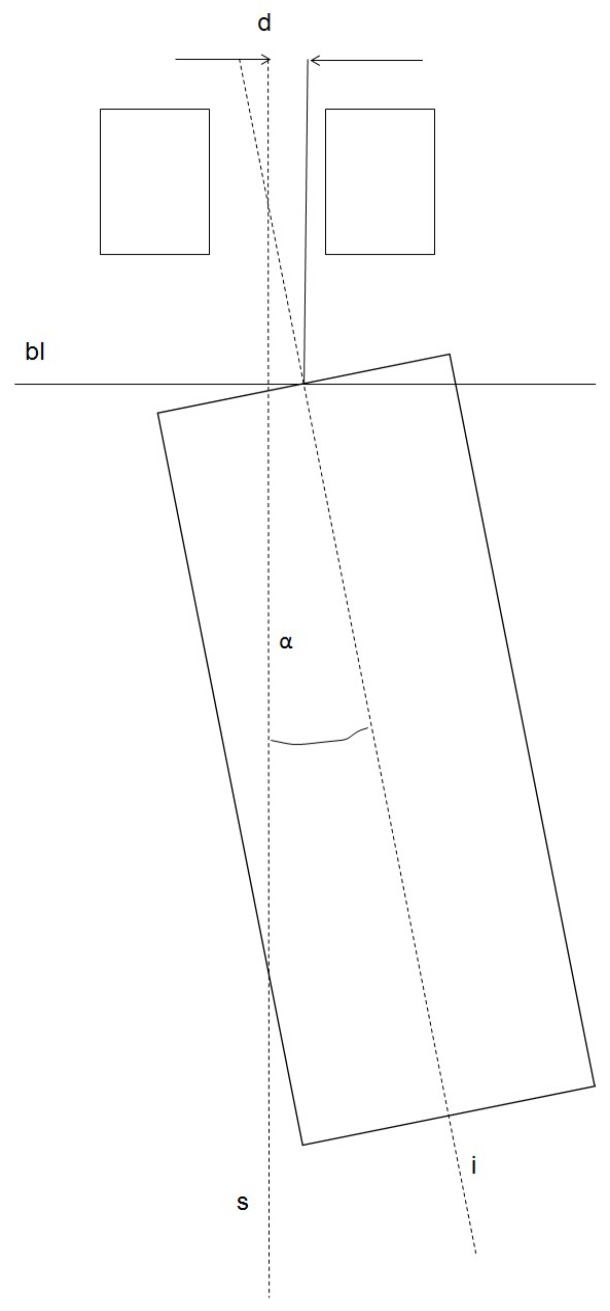
Two-dimensional schematic drawing of the measurements. The angle α between the median axis of the sleeve (s) and the median axis of the implant (i) was measured. Furthermore, the mismatch (d) between the planned median axis of the sleeve (s) and the median axis of the implant (i) was assessed at the bone level (bl). All measurements were likewise performed in the oro-vestibular and mesio-distal direction.

The i-Dixel OneVolumeViewer 2.6.0. software (J. Morita MFG. CORPORATION, Kyoto, Japan) was used for the evaluation. For measuring, the implant region was depicted in the axial, sagittal, and coronal plane at a zoom adjustment of 400%. In order to assess the accuracy of the real implant position compared to the planned implant position, different parameters were recorded. The longitudinal median axis of the sleeve and the longitudinal median axis of the implant were used. The angle between the longitudinal axes and the mismatch of the axes of the implant and the pilot sleeve at the bone level (coronal part of the implant) in the oro-vestibular as well as in the mesio-distal direction were assessed (Figure 2). In order to assess the direction of the mismatch, different algebraic signs were used: in a case where the deviation was to the mesial or oral direction, it was marked with a “+“. When the deviation was to the distal or vestibular direction, it was marked with a “−”. Furthermore, the distance in mesio-distal direction to the adjacent teeth and the vertical distance between the implant shoulder and the artificial jaw “bone” level were determined. All parameters were measured three times and the arithmetic means were calculated. The values were captured in an Excel^®^ chart (Microsoft Inc., Redmond, WA, USA).

The statistical analysis was performed using SPSS Statistics 24 (IBM Corporation, Armonk, NY, USA). The mean values and their standard deviations, as well as the median values, were calculated. The non-parametrical Wilcoxon rank tests were performed for the pair-wise comparisons due to the fact that the values had no normal distribution. The Mann-Whitney-U test was applied for the inter-group comparisons. For statistical significance, a level of α = 0.05 was stated.

## 3. Results

### 3.1. Demographical Data and Education

A total number of 61 dental students completed the study. The mean age of the participants was 26 years (ranging from 23 to 35 years). The majority of the participating persons stating their handedness was right handed (50 right-handed vs. 5 left-handed). Out of the 61 dental students, 15 had completed professional education prior to joining dental school. Twenty-four dental students had participated in dental implant training before. The details of the demographical and educational data are shown in Table 1.

### 3.2. Deviation of Implant Axis and Implant Position—Orientation Template vs. Free-Hand Implant Insertion

Detailed data and the *p*-values of the pair-wise comparisons are listed in Table 2. When considering the distance from the implant shoulder to the bone level of the artificial jaw, it was obvious that the dental students were inserting the implant closer to the ideal vertical position when performing free-hand implant insertion.

A statistically significant difference between the orientation template and free-hand implant insertion could be observed (*p* = 0.004). When comparing the accuracy achieved by the use of the orientation template to the free-hand implant insertion, it was higher for the deviation in the mesial-distal direction and for the mismatch in the oro-vestibular direction. For the coronal mismatch in the oro-vestibular and mesio-distal directions, a statistical significance was reached (*p* < 0.001; *p* = 0.049). For the other values, the accuracy considering the planned implant position was slightly higher for free-hand implant insertion. On the other hand, in the free-hand implant insertion group, a perforation to the buccal lamella could be observed in 16 cases. Furthermore, four dental students perforated the lingual lamella. In contrast, a perforation to the buccal lamella occurred only in one case when using the orientation template.

Generally, there was found no statistically significant difference in accuracy between sex, age, professional education, or year of training. Only single parameters in the comparisons (e.g., deviation of the parallel axis to the adjacent tooth/implant in the free-hand implant insertion) differed when statistically analyzing the above-mentioned parameters.

### 3.3. Attended Time for Implant Insertion

The mean time for implant insertion using the orientation template was 9.04 min, compared to 9.41 min for the free-hand implant insertion. When comparing the attended time for the guided implant insertion to the free-hand implant insertion, no statistically significant difference was found (*p* = 0.217). Interestingly, there was a difference between right- and left-handed participants for the time needed to insert the implants. The mean value of the attended time for right-handed participants was 8.89 min, while for left-handed participants it was 11.75 min. However, no statistically significant differences were obvious for the guided implant insertion (*p* = 0.064) nor for the free-hand implant insertion (*p* = 0.875).

## 4. Discussion

In the current examination, the accuracy achieved in the deviation of the implant axis compared to the sleeve axis was slightly higher for the orientation template in the mesio-distal direction (0.67 ± 3.48° vs. 1.34 ± 5.15°). In the oro-vestibular direction, the free-hand implant insertion was closer to the pre-planned position than the orientation template (0.71 ± 3.48 vs. 2.28 ± 2.75). One reason for this might be the flexibility of the orientation template made by thermoforming. The application of rigid plotted templates could have been advantageous as a smaller deformability is achieved [1]. Another reason might be because orientation templates only determine pilot cavity. When preparing the implant cavity, minor changes in angulation are still possible. This setback might be overcome by the use of plotted full-guiding templates, reducing the possibility of deviating from the intended direction, which might be an advantage particularly for inexperienced operators. However, in a study performed with thirteen patients comparing different manufacturing methods for the templates, a mean deviation of 3.5° for the implant axis was found [1]. This value can be considered as comparable to our findings. In a recent review, the range for the angular deviation for different types of implant templates and planning software was between 1.09 ± 0.51° and 7.25 ± 2.67° [15]. For in vitro studies performing the full guided implant insertion, angle deviations between 1.09 ± 0.51° and 4.71 ± 1.63° were reported [16,17,18]. Comparing those findings to the accuracy achieved in the present examination, our values are in the range of the previous results. In one study, the performing surgeons were experienced [17], and in another, experienced and inexperienced surgeons were compared [18]. It was concluded that experienced surgeons were able to place the implants more accurately. In the current study, all participants can be considered inexperienced as they had placed less than 10 implants at the time of the examination [18]. However, considering the angle, the accuracy level achieved was in the range of the mentioned studies. A finding which differed from the other studies was the considerably high standard deviation found in our current examination. A reason for this might have been the fact that in the mentioned studies, multiple implant insertions were performed by a small number of participants. In our study, one implant insertion per method was performed by one individual, resulting in higher mean variation.

The coronal mismatch found between the planned and the achieved implant position ranged from −0.22 ± 0.62 mm (distal direction) to 0.17 ± 0.41 mm (mesial direction) for the orientation template. In the mesio-distal direction, free-hand implant insertion was slightly more accurate than by using an orientation template. One reason for the higher mismatch when using the orientation template might have been that the area for the implant placed using the orientation template was a free-end gap. Thus, the template was distally unsupported by a tooth, possibly resulting in a higher tilting mobility of the template made by thermoforming. Thus, a little mismatch to the desired implant position is likely when inserting the pilot drill. The same deformation was observed in a study using templates made by vacuum forming in mandibles with a free-end gap [7]. The use of a plotted template would have shown an advantage compared to templates made by thermoforming, due to the higher rigidity. In a recent study, Matta et al. reported that the fabrication method had an impact on the three-dimensional accuracy of implant placement [1]. They found a statistically significant lower three-dimensional precision for templates made by thermoforming compared to a printed guide. However, they concluded that there was no clinical impact, due to the differences being in the decimillimeter area [1]. In our study, the differences were in the same range, i.e. in the decimillimeter area, meaning the results can be considered as comparable.

In the oro-vestibular direction, the mean mismatch measured for the dental students was 0.24 ± 0.62 mm. Thus, a statistically significant lower mismatch was achieved with the orientation templates compared to the free-hand implant insertion. In a recent study, Dimitrijevic et al. observed that the majority of the participants in a test containing a distance estimation task overestimated the distance, which might lead to an implant insertion not being in the desired position when having no guidance available [13]. From this point of view, the application of an orientation template might lead to an advantage in determining the implant position. In a clinical study, the use of an implant guide in a maxillary model led to a statistically significant less coronal lateral deviation compared to the free-hand implant insertion [4]. In contrast, the mesio-distal mismatch found in our study for the free-hand implant insertion group (front area) was slightly less than in the implant positions placed using the orientation template. One reason for this might be that the implant position for the free-hand implant insertion was located in the front area bordered by two teeth, thus allowing easier access and better orientation than in the distal gap. Additionally, the application of orientation templates enables a minor change in direction indicated by the pilot-drill. For inexperienced participants, this might lead to an unintentional change in the direction and position of the desired implant cavity due to a lack of experience handling surgical equipment and when employing it for the first time. In order to avoid this, the use of plotted full-guiding templates, which are more rigid, might be advantageous for inexperienced dentists. Vermeulen stated that even experienced clinicians benefit from using a plotted three-dimensional implant guide when inserting an implant in the frontal maxillary area, compared to the free-hand insertion [4]. In contrast to our study, jaw models mounted in dummy heads were used in his study, more closely resembling a clinical situation than in the presented study. Thus, the visibility and accessibility might have been more difficult compared to our study, probably resulting in lower accuracy levels for the free-hand implant insertion. One setback for the reproducibility of the results is the fact that the implants were placed in different regions of the jaw. The implant placed using the orientation template was in the right side of the mandible, whereas the implant to be placed free-hand was in the more accessible front area. The implant position in the front was chosen to allow the participants better orientation. As the frontal gap was an inter-dental space, the adjustment of the implant position and implant inclination was more easily achievable when having two adjacent teeth as a visual guide. However, an implant position in the area of a premolar tooth on the contra-lateral side of the mandibular model would have allowed a more sufficient comparison of the methods applied in our study.

In general, no statistically significant difference could be found for the individual factors, e.g. sex, age, or prior professional education. This might be due to the wide variance in participants and the relatively small sample size considering those factors. Furthermore, a statistically significant difference regarding accuracy between left- and right-handed participants could not be observed in the present examination. In contrast, van de Wiele et al. reported that implants placed by a right-handed surgeon on the right side of the patient were more accurate than on the left side [19]. This was not the subject of the examination in the present study, as both implants were placed in the lateral right or the frontal area of the model.

The laboratory implant course is integrated into the fifth-year curriculum. An optional hands-on course, in which 24 students had participated before, is offered in the fourth year. The design of a laboratory course was chosen due to the fact that for the majority of the participants, it was their first hands-on experience regarding dental implants. As implant insertion involves potential risks and complications for the patients, e.g. a lesion of the inferior alveolar nerve, training should be provided in a preclinical situation. Luck et al. observed an improvement in fine motor skills in dental students which they explained was due to continuous dexterity training [20]. However, a variation between the participants in manual dexterity was obvious. The application of orientation templates might be useful even in a laboratory setting to give students support with estimations of the right distance relating to the adjacent tooth and the oro-vestibular position. Additionally, despite some of the short-comings, some points are expedient when performing the set-up as a laboratory study for students. A three-dimensional radiographic analysis of the planned and realized implant position is not possible in a clinical set-up, due to the Radiation Protection Ordinance. With respect to radiation protection, it would not be justifiable to perform three-dimensional radiographic examination when analyzing the three-dimensional position of the implants. Furthermore, by performing this examination in an undergraduate laboratory course, it is possible to give the students feedback on their performance in regard to their level of accuracy. This might be a step in the direction of quality-controlled training, which also better ensures patients’ safety [9].

When analyzing the time required for implant insertion, it was obvious that the dental students needed more time when performing free-hand implant insertion. One reason for this might be the increased need of control when positioning the implant according to the adjacent teeth. However, the difference in time had no statistical significance.

An interesting finding was the fact that left-handed participants required more time when performing implant insertion. One reason for this might be the use of right-handed equipment in this examination. Furthermore, the instructors were right-handed and demonstrated the sequences of implant insertion using their right hand. Thus, the mental application of the sequence might have been easier for the right-handed rather than for the left-handed participants, resulting in more time being required for implant insertion for the left-handed participants. In a recent examination, it was reported that left-handed dental students had problems when being instructed by right-handed tutors [21]. This, however, was not assessed in our examination. When looking at the distribution of handedness, the percentage of participants stating left-handedness in the present study was about 10.6 percent. This is comparable to the percentage found in another study among dentists [21,22]. However, this percentage may vary widely, between around 2 and 30 percent [23].

## 5. Conclusions

When looking over the results of our study, our initial hypothesis could be considered as being partly fulfilled. The deviation in mesio-distal direction and the mismatch in oro-vestibular direction could be reduced by applying orientation templates, whereas the deviation in oro-vestibular direction and the mismatch in mesio-distal direction was slightly higher compared to free-hand implant insertion. However, the deviations found in our study were comparable with those found in other recent studies. In relation to time taken, the application of orientation templates was advantageous. Further studies examining the three-dimensional accuracy of placing dental implants by undergraduate dental students with the help of rigid full-guided templates would be interesting, and may eliminate the shortcomings caused by orientation templates made by thermoforming.

## Figures and Tables

**Table 1:** Demographical data of the participants in regard to age, gender, and handedness. Furthermore, optionally professional training in the group of the dental students is listed (man.—manual; req.—required). In cases where no specification was made by the student, it was marked as “not stated”.

Parameter	Group	Dental Students
age	18–30 years	54
	> 30 years	7
gender	female	40
	male	15
	not stated	6
handedness	left	5
	right	50
	not stated	6
professional education before dental school	yes	15
	no	38
	not stated	8
type of professional training	high man. fine motor skills	4
	medium man. fine motor skills	7
	no man. fine motor skills req.	4

**Table 2:** Pair-wise comparison of the parameters between the use of the orientation template and free-hand implant insertion. The mean values, median values, standard deviations of the mean values (SD), and the *p*-values are depicted. A “+” means a deviation in mesial or oral direction. A “−“ refers to a deviation in distal or vestibular direction. Statistically significant differences are marked in bold font.

Parameter	Group	Mean	± SD	Median	Min	Max	*p*-Value
distance implant shoulder/	orientation template	0.57	0.36	0.53	−0.29	1.70	0.004
bone in mm	free-hand	0.41	0.43	0.38	−0.96	1.60	
deviation implant/sleeve	orientation template	−0.67	3.48	0.14	−12.14	7.26	0.304
mes-dis in degrees	free-hand	−1.34	5.15	−0.17	−10.21	9.36	
deviation implant/sleeve	orientation template	2.28	2.75	2.45	−3.79	10.83	0.057
oro-ves in degrees	free-hand	0.71	3.99	0.83	−14.52	15.10	
mismatch implant/sleeve	orientation template	−0.22	0.62	−0.33	−1.62	1.23	0.049
mes-dis in mm	free-hand	0.06	0.79	0.31	−1.85	1.12	
mismatch implant/sleeve	orientation template	0.24	0.62	0.31	−1.89	1.17	<0.001
oro-ves in mm	free-hand	−1.08	0.55	−1.13	−2.10	0.81	
deviation of parallel axis	orientation template	5.49	3.34	5.21	0.36	12.14	<0.001
tooth/implant in degrees	free-hand	2.01	2.41	1.20	0.07	15.45	
time attended for implant	orientation template	9.04	4.63	8.00	1	25	0.217
insertion in minutes	free-hand	9.41	3.77	9.00	3	20	
